# Polycomb CBX7 Directly Controls Trimethylation of Histone H3 at Lysine 9 at the *p16* Locus

**DOI:** 10.1371/journal.pone.0013732

**Published:** 2010-10-29

**Authors:** Qiang Li, Xiuhong Wang, Zheming Lu, Baozhen Zhang, Zhenpo Guan, Zhaojun Liu, Qiming Zhong, Liankun Gu, Jing Zhou, Budong Zhu, Jiafu Ji, Dajun Deng

**Affiliations:** 1 Key Laboratory of Carcinogenesis and Translational Research (Ministry of Education), Department of Etiology, Peking University Cancer Hospital and Institute, Beijing, China; 2 Department of Internal Medicine, Peking University Cancer Hospital and Institute, Beijing, China; 3 Department of Surgery, Peking University Cancer Hospital and Institute, Beijing, China; National Institute on Aging, United States of America

## Abstract

**Background:**

H3K9 trimethylation (H3K9me3) and binding of PcG repressor complex-1 (PRC1) may play crucial roles in the epigenetic silencing of the *p16* gene. However, the mechanism of the initiation of this trimethylation is unknown.

**Methodology/Principal Findings:**

In the present study, we found that upregulating the expression of PRC1 component *Cbx7* in gastric cancer cell lines MGC803 and BGC823 led to significantly suppress the expression of genes within the *p16*-*Arf-p15* locus. H3K9me3 formation was observed at the *p16* promoter and Regulatory Domain (RD). CBX7 and SUV39H2 binding to these regions were also detectable in the CBX7-stably upregulated cells. CBX7-SUV39H2 complexes were observed within nucleus in bimolecular fluorescence complementation assay (BiFC). Mutations of the chromodomain or deletion of Pc-box abolished the CBX7-binding and H3K9me3 formation, and thus partially repressed the function of CBX7. SiRNA-knockdown of *Suv39h2* blocked the repressive effect of CBX7 on *p16* transcription. Moreover, we found that expression of CBX7 in gastric carcinoma tissues with *p16* methylation was significantly lower than that in their corresponding normal tissues, which showed a negative correlation with transcription of *p16* in gastric mucosa.

**Conclusion/Significance:**

These results demonstrated for the first time, to our knowledge, that CBX7 could initiate H3K9me3 formation at the *p16* promoter.

## Introduction

Epigenetic silencing is the main way for inactivation of tumor suppressor genes such as *p16* (*Ink4a*, *CDKN2A*) in numerous human cancers [1]. Both DNA methylation and histone modifications could suppress transcription of these genes. Epigenetic inactivation of *p16* is an intensively studied event, which plays a role in carcinogenesis [2]–[4]. Unfortunately, the mechanisms of epigenetic inactivation of such genes including *p16* are still largely unknown.

Expression of *p16* is regulated by a number of transcription factors, including activator Ras, Myc, TGFβ, ETS2, and silencer pRB, Polycomb group (PcG) proteins such as EZH2, BMI1, CBX2, CBX7, CBX8, etc [5]. Different Polycomb group complexes regulate common target genes [6]. For instance, PcG proteins suppress expression of *Homeobox* genes and the *p16-Arf-p15* locus mainly through the Polycomb repressive complex-1 and -2 (PRC1 and PRC2), which control cell fate and cancer development [6]–[9]. It is well known that binding of PRC2 with the *p16* promoter can initiate reversible repression of its transcription through EZH2-mediated trimethylation of histone H3 at lysine 27 (H3K27me3) [10]–[12]. Unlike the reversible H3K27me3, trimethylation of histone H3 at lysine 9 (H3K9me3) is an epigenetic landmark of the eukaryotic genome [13]–[15]. Some PRC1 members such as CBX7 bind to H3K9me3 within both heterochromatin and silenced euchromatic loci *in vitro*, which might play a role in the epigenetic maintenance of transcriptional silence of target genes [16]. SUV39H1, SUV39H2, and G9a catalyze trimethylation of H3K9 in mammalian cells [17]–[19]. It is reported that CBX7 suppresses transcription of *p16* in human fibroblast cells [20], but how CBX7 represses the transcription is not clear. In the present study, we first report, to our knowledge, that the PRC1 member CBX7 could initiate the formation of H3K9me3 at the *p16* promoter and thus promote the repression of P16 expression through a SUV39H2/G9a-dependent pathway.

## Results

### Upregulation of CBX7 suppresses transcription of *p16* through initiation of H3K9 trimethylation

**Figure 1:**
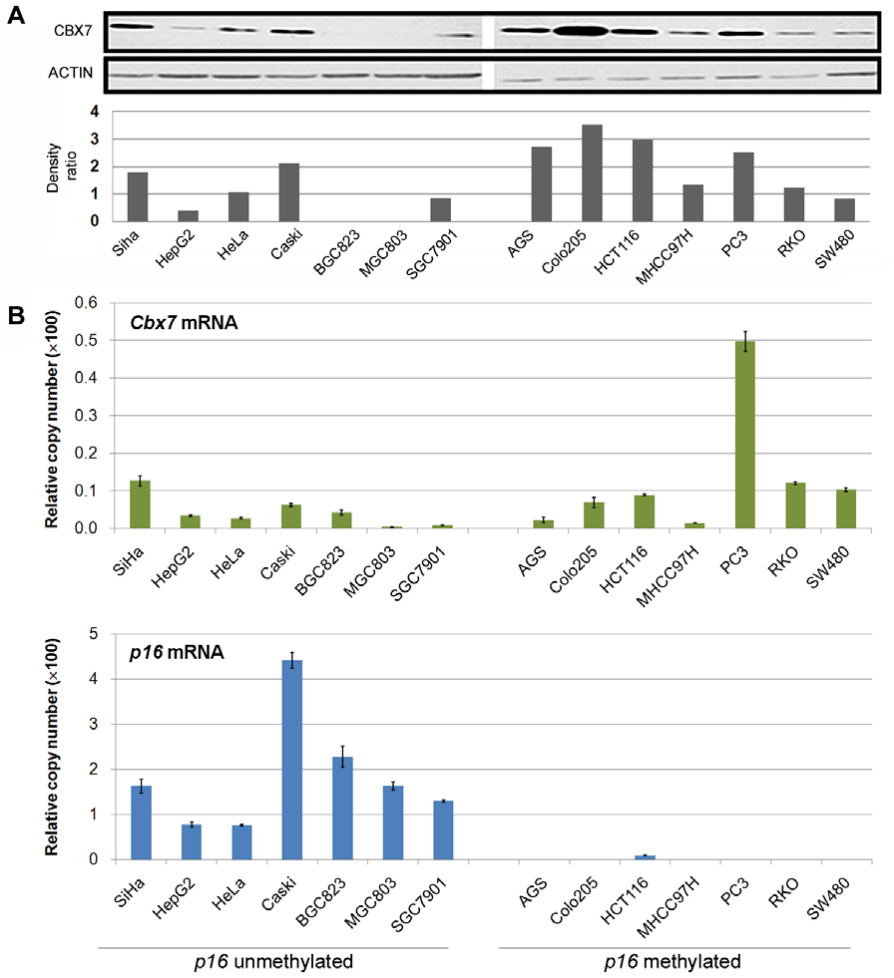
CBX7 expression and transcription of *Cbx7* and *p16* in human cancer cell lines. (**A**), analysis of CBX7 expression by Western blot. (**B**) analysis of of *Cbx7* (up) and *p16* (down) expression by quantitative RT-PCR. The methylation status of *p16* CpG island is marked according to the results displayed on Figure S1.

To select suitable cell lines to investigate the role of CBX7 in epigenetic inactivation of *p16*, we analyzed the methylation status of *p16* and the mRNA levels of *Cbx7* and *p16* in 14 human cell lines (Figure 1 and Fig. S1). *p16* Methylation was observed in 6 cell lines including PC3 and HCT116, but not in the gastric cancer cell lines MGC803 and BGC823. Thus, these cancer cell lines MGC803, BGC823 (*p16*-active and low level of CBX7 expression), and PC3 (*p16*-methylated and high level of CBX7 expression) were used as the representative cell lines in the subsequent experiments.

**Figure 2:**
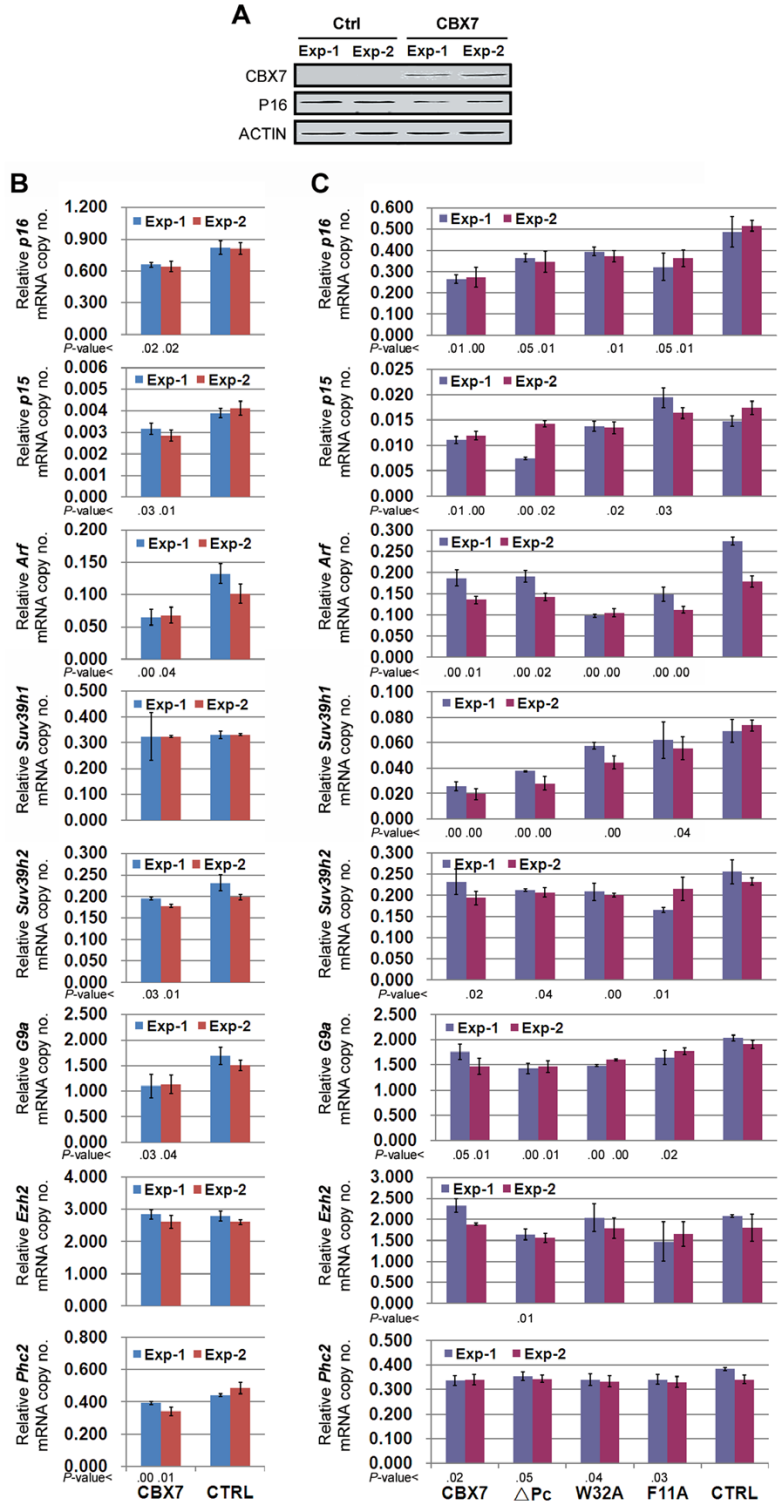
Effects of CBX7 on expression of the *p16*-*Arf*-*p15* locus, HMTases, and genes at other loci. Results of two independent experiments were displayed. (**A**), analysis of expression of *Cbx7* in MGC803 cells 72 hours after transfection by Western blot assays. (**B**), analysis of transcription level of genes located within the *p16-Arf-p15* locus, two control genes (*Ezh2* and *Phc2*) located at other loci and three HMTases, in BGC823 cells 72 hours after transfection by quantitative RT-PCR. (**C**), analysis of transcription level of genes located within the *p16-Arf-p15* locus, two control genes (*Ezh2* and *Phc2*) located at other loci and three HMTases, in MGC803 cells 72 hours after transfection by quantitative RT-PCR. *P*-value less than 0.05 for CBX7/mutants vs. CTRL was listed below each column, respectively. Each column represents the average value of triplicate. The STDEV value was on the top of column. CTRL, cells transfected with the pcDNA3.1(+)/myc-His A control vector; CBX7, cells transfected with the vector containing the full coding region of wildtype *Cbx7*. ΔPc, Pc-box deleted CBX7; F11A and W32A, CBX7 containing F11A and W32A mutation within chromodomain, respectively.

To set up models for silencing *p16* transcription by CBX7, we transiently transfected the wildtype *Cbx7* expression vector into MGC803 and BGC823 cells. Expression of *p16* decreased in both cell lines at 72 hours after transfection (Figure 2). Expression of *Arf* and *p15* located within the same locus also decreased (Figure 2B and 2C). These results suggest that CBX7 can repress the expression of the *p16-Arf-p15* locus in these cell lines. Moreover, weak downregulation of histone methyltransferase (HMTase) gene *G9a* was observed in both cell lines 72 hours after CBX7 transfection. Although transcription of *Suv39h1* was drastically reduced in MGC803 cells, consistent downregulation was not observed in BGC823 cells. The levels of *Suv39h2* and *Ezh2* mRNA were not affected in both cell lines. Transcription of *Phc2* was downregulated, although to a weak extent (Figure 2B and 2C).

It is reported that the Pc-box within CBX7 is essential for its binding to Ring1 and this impairs its ability to repress *p16* transcription [20], [21]. Mutations in critical residues of the chromodomain (F11A and W32A, Figure S2) or deletion of the Pc-box (ΔPc) inhibited the ability of CBX7 to extend the life span of cells [20]. In the present study, we also found that the deletion of Pc-box or chromodomain mutations only partially abolished the repressive effect of CBX7 on transcription of *p16* in the transiently transfected cell lines. Moreover, the chromodomain mutations abolished the downregulation of *Suv39h1* in MGC803 cells significantly. But deletion of the Pc-box had no effect on downregulation of *Suv39h1* and *p15*. Downregulation of *Arf* was affected by the chromodomain mutation F11A (Figure 2C).

**Figure 3:**
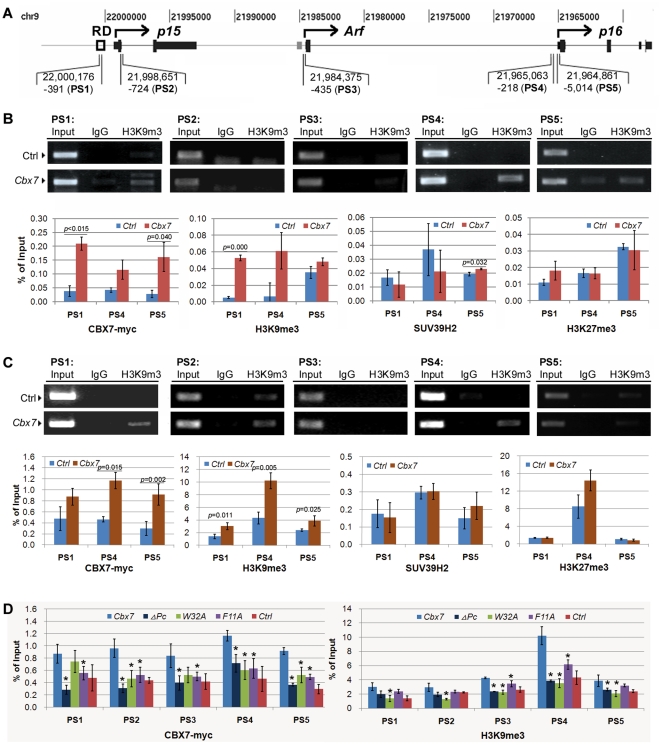
H3K9me3 formation, CBX7 and SUV39H2 binding within *p16* and Regulatory Domain regions in ChIP assays. (**A**), locations of the *p15-Arf-p16* locus at chromosome 9 and the corresponding fragments amplified with primers (PS1∼5) used in ChIP assays (based on web http://genome.ucsc.edu, Mar. 2006, assembly). (**B**) and (**C**), analysis of H3K9me3 formation by ChIP assay in cell line MGC803 and BGC823 transiently transfected with *Cbx7* expression vector, respectively. H3K9me3 formation, CBX7- and SUV39H2-binding were also analyzed by quantitative ChIP assays in these cell lines. (**D**), results of CBX7-binding and H3K9me3 formation by ChIP assays in BGC823 cells 72 hours after transfected with the wildtype and mutant *Cbx7*-vectors. *, versus the wildtype CBX7, *P*<0.05; Ctrl, cells transfected with the control vector pcDNA3.1(+)/myc-His A; Cbx7, cells treated with the vector encoding full length of coding region of the wildtype CBX7; ΔPc, Pc-box deleted CBX7; F11A and W32A, CBX7 containing F11A and W32A mutation within chromodomain, respectively; % of Input, the percentage of the average relative copy number of the tested triplicates to that of Input control.

H3K9me2, H3K9me3 and H3K27me3 are well-known epigenetic markers for gene repression. The Regulatory Domain (RD) is reported previously to play an important role in the regulation of *p16-Arf-p15* locus [22]. To address whether histone modifications are involved in the repression induced by CBX7, we analyzed H3K9me3 at the *p16-Arf-p15* locus and RD using chromatin immunoprecipitation assay (ChIP). H3K9me3 was seen within the RD and the *p16* promoter and 5′UTR regions in the *Cbx7* transiently transfected MGC803 and BGC823 cells (Figure 3B and 3C: PS1, PS4, and PS5). Formation of H3K27me3 was not increased within these tested loci (Figure 3C). H3K9me2 was not detectable either (data not shown). Binding of CBX7 mutant proteins to five tested DNA fragments within the *p16-Arf-p15* locus was not detectable and formation of H3K9me3 was not induced either (Figure 3D).

**Figure 4:**
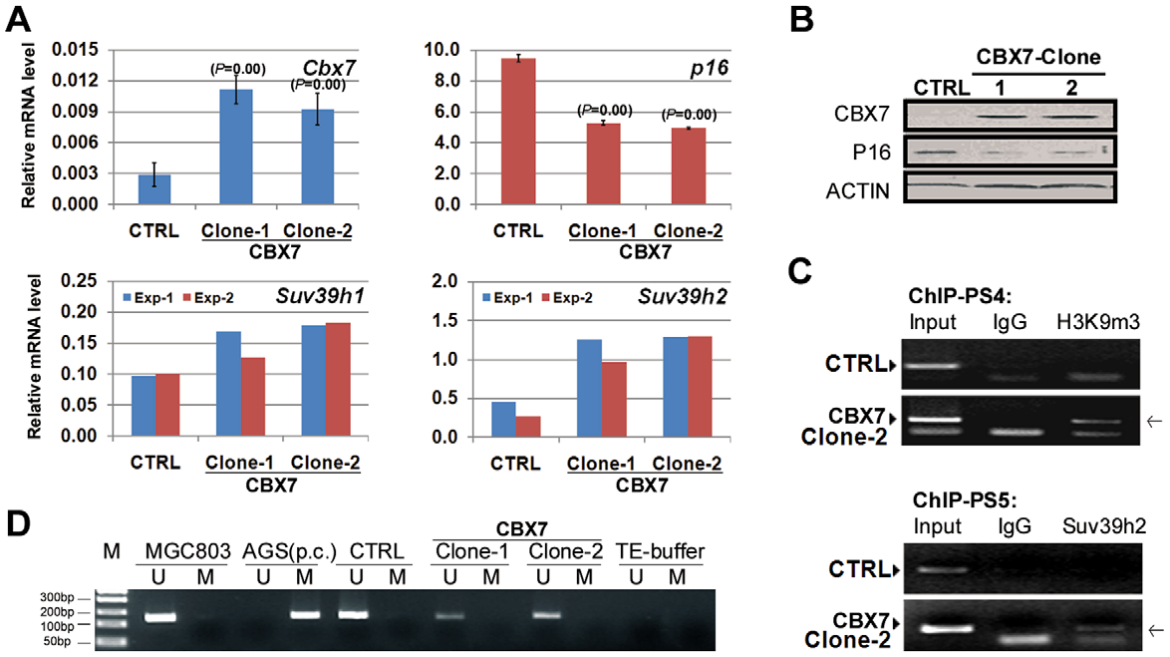
HMTase expression, H3K9me3 formation, SUV39H2 binding, DNA methylation within *p16* CpG island. (**A**), analysis of expression of *Suv39h1* and *Suv39h2*, *p16*, and *Cbx7* in two CBX7-stably transfected MGC803 subclones by quantitative RT-PCR. (**B**), analysis of CBX7 and P16 expression in the CBX7-stably transfected subclones by Western blot. (**C**), ChIP analysis of H3K9me3 formation within the *p16* promoter and recruitment of SUV39H2 to the 5′UTR of *p16* gene in Clone-2. The target PCR product is marked by an arrow. (**D**), analysis of *p16* methylation by methylation-specific PCR (MSP). Genomic DNA of AGS cell line was used as positive control (p.c.) for *p16* methylation in the MSP assay. CTRL: MGC803 cell stably transfected with pcDNA3.1(+)/myc-His A control vector; Clone-1/-2: the two MGC803 subclones stably transfected with vector containing the full coding region of wildtype *Cbx7*; Exp-1/-2: two independent experiments.

In the CBX7-stably transfected MGC803 subclone(s), in which downregulation of *p16* expression is maintained, expression of *Suv39h1*and *Suv39h2* increased significantly (Clone-1 and Clone-2; Figure 4A and 4B). The H3K9me3 formation was shown within the tested *p16* promoter (Figure 4C: PS4), though *p16* methylation was not observed in these subclones even at passage 80 by methylation-specific PCR (MSP) (Figure 4D: Clone-1 and Clone-2).

**Figure 5:**
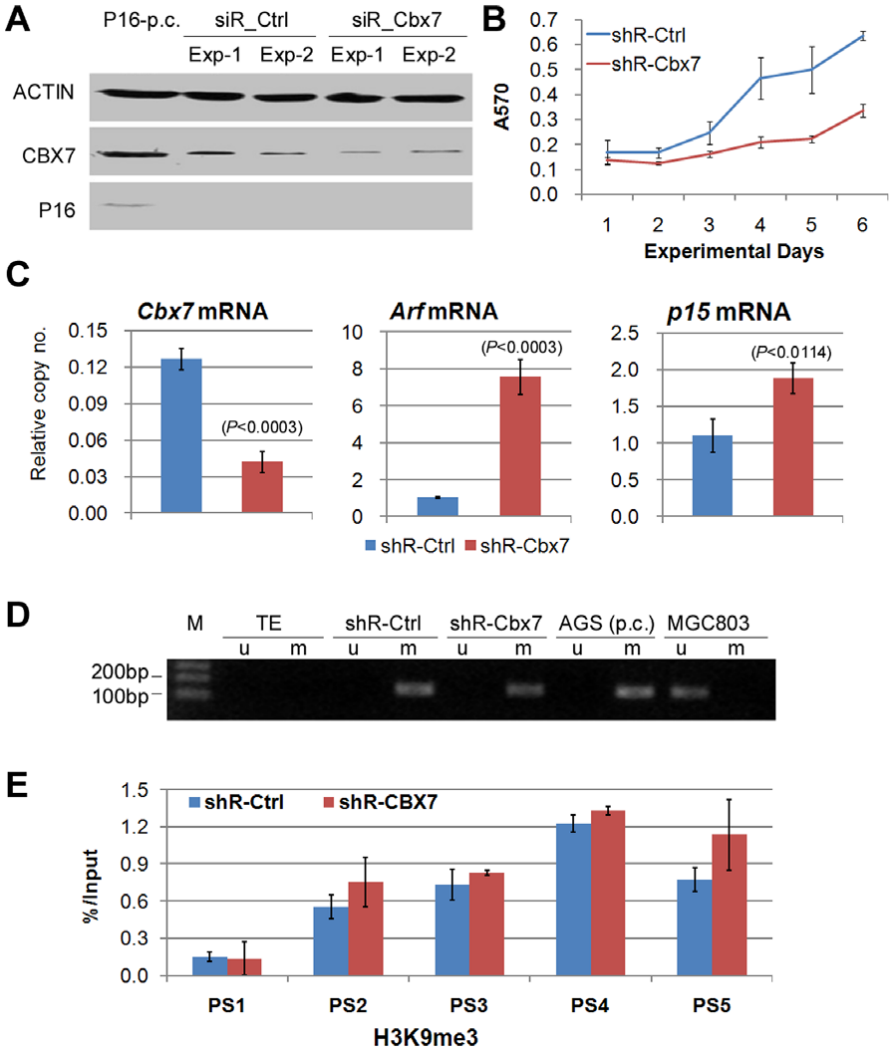
Effect of downregulation of *Cbx7* on expression of *p16*/*Arf*/*p15*, cell growth and *p16* methylation. (**A**), analysis of CBX7 expression in PC3 cells by Western blot assay 72 hours after CBX7 knockdown. Protein extracted from 293T cells was used as P16 positive control (P16-p.c.). (**B**), the growth curves of PC3 cells stably transfected with the scramble control shRNA vector (shR-Ctrl) or shRNA-Cbx7 vector (shR-Cbx7) in MTT assay. (**C**), analysis of transcription of *Cbx7, Arf*, and *p15* by quantitative RT-PCR. The statistical significance of the differences of mRNA relative copy number between cells stably transfected with shR-Ctrl and shR-Cbx7 were labeled above the top of one of the columns. (**D**), analysis of *p16* methylation by MSP in the *Cbx7* stably knockdown PC3 cells. Genomic DNA from AGS and MGC803 cell lines was used as positive control of methylated and unmethylated *p16* respectively. (**E**), comparison of H3K9me3 level by ChIP assays within various locations as illustrated in Figure 3A.

In addition, transient knockdown of CBX7 by siRNA did not reactivate P16 expression in *p16*-methylated PC3 cells (Figure 5A). Stably knockdown of CBX7 by shRNA inhibited the growth of PC3 cells (Figure 5B), but induced neither *p16* transcription (Figure 5C) nor demethylation of *p16* CpG island (Figure 5D). Significant change of H3K9me3 level at the five tested fragments was not observed (Figure 5E). However, significant upregulation of the transcription of *Arf* and *p15* was observed in PC3 cells (Figure 5C).

### SUV39H2 and G9a contribute to H3K9 trimethylation within the *p16* 5′UTR induced by CBX7

**Figure 6:**
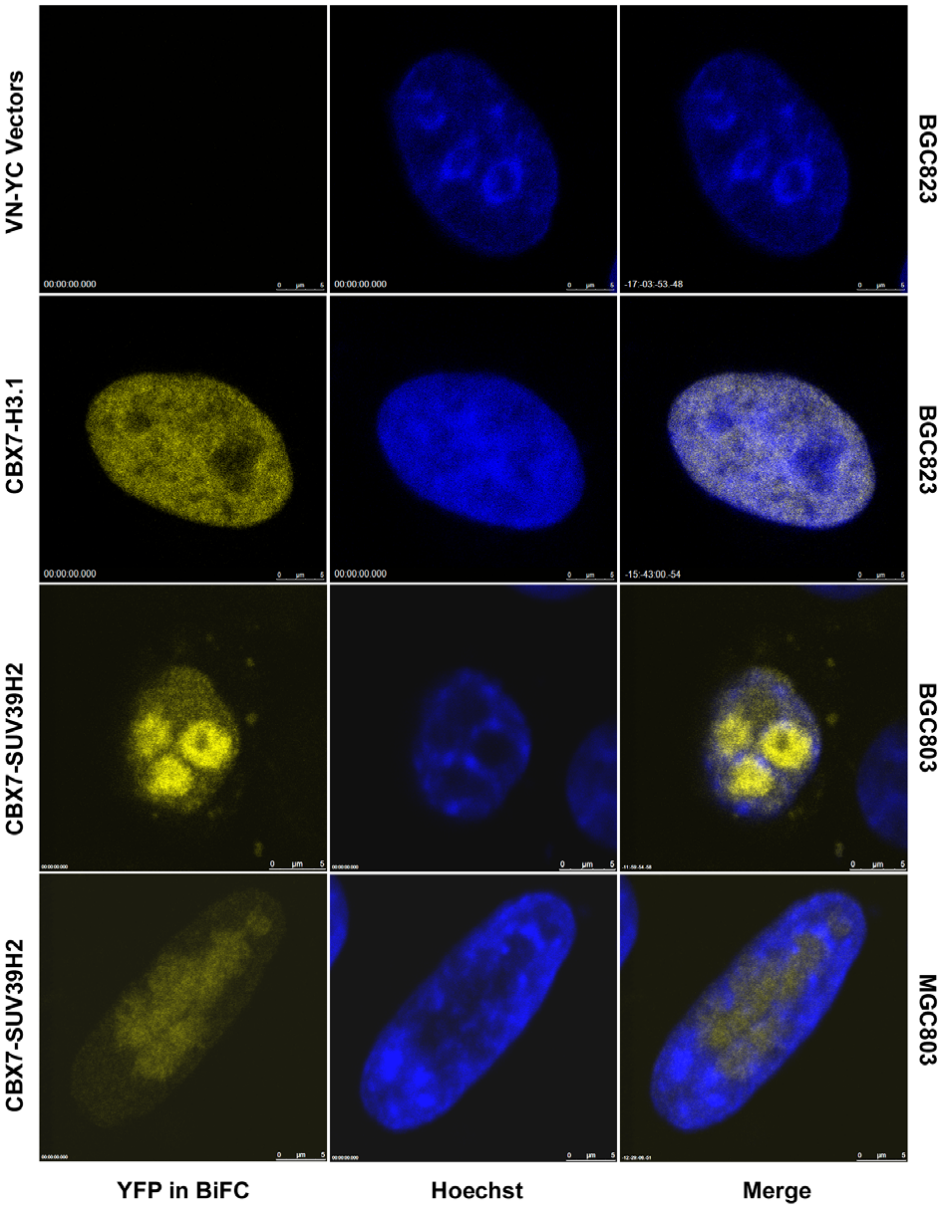
Distribution of CBX7-SUV39H2 complexes in cancer cell line BGC823 in bimolecular florescence complementation (BiFC) assay. 36 hours after transfection, images of the BiFC complex of CBX7-SUV39H2 (yellow) and Hoechst (blue) fluorescence were obtained and merged. The blank VN- and YC-vectors were used as negative controls. VN-CBX7 and YC-Histone 3.1 (H3.1) vectors were used as positive controls.

G9a, SUV39H1 and SUV39H2 are three histone methyltransferases (HMTases) involved in trimethylation of H3K9. Hence, we studied which HMTase was the enzyme responsible for the trimethylation of H3K9 after CBX7 upregulation. We initially analyzed the binding of SUV39H2 with *p16-Arf-p15* locus by ChIP assay, and found that SUV39H2 was weakly recruited to the *p16* 5′UTR in cell lines transiently transfected with CBX7 (Figure 3B and 3C), but strongly in the tested CBX7-stably upregulated subclone-2 (Figure 4C). Thus, bimolecular fluorescence complementation assay (BiFC) was further used to detect the possible interaction between CBX7 and SUV39H2. Amino acids 173–238 of YFP (YC) were fused to the C-termini of SUV39H2 to construct the YC-*Suv39h2* vector. The VN-*Cbx7* vector, containing the N-terminal 172 amino acids of Venus (VN) fused to the N-termini of CBX7 protein, was also used in the BiFC assay, because it remains an active CD domain with high affinity to Histone 3.2 and 3.1 [23]. Images of the BiFC complex of CBX7-SUV39H2 (yellow) and Hoechst (blue) fluorescence were obtained in BGC823 (Figure 6) and MGC803 cells 36 hours upon transfection (Figure S3). Interaction between CBX7 and SUV39H2 was mainly observed within the nucleus, especially the Hoechst-negative staining areas in both cell lines (Figure 6 and Figure S3). Such interaction could not be observed in the tested cells transfected with blank VN- and YC-vector controls. Interaction between CBX7 and Histone 3.1 positive control was mainly observed within the Hoechst-positive staining area (Figure 6). At the same time, we cloned *Cbx7* into the pEFGP-C1 vector and transfected it to MGC803 cells and found that CBX7 was distributed in the entire nucleus with higher concentration at the Hoechst-negative staining areas (Figure S3), which was consistent with the BiFC results. Although CBX7-Histone 3.1 complexes were observed in Co-IP assay, CBX7-SUV39H2 complexes were not detected (Figure S4).

**Figure 7:**
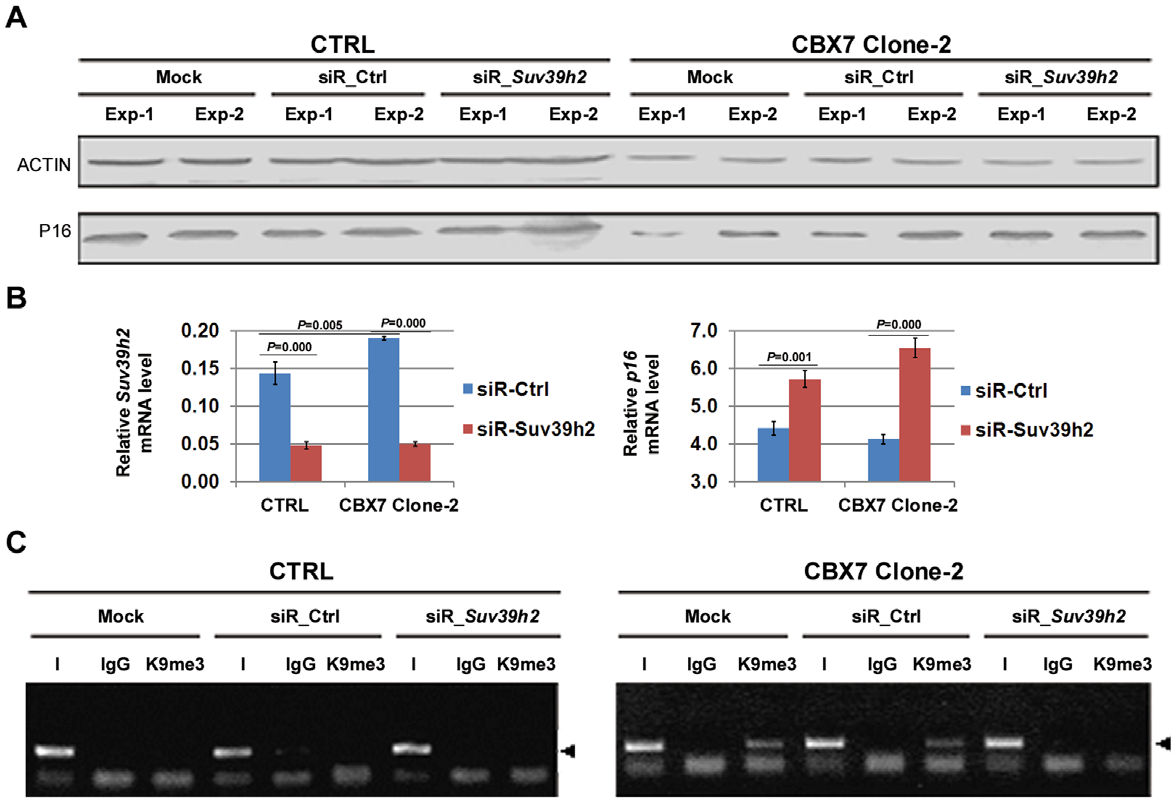
Effect of siR-Suv39h2 on *p16* expression, H3K9me3, and binding of SUV39H2 within the *p16* 5′UTR. The CBX7-stably transfected MGC803 subclone CBX7 Clone-2 was analyzed 72 hours after knockdown of *Suv39h2* by siRNA. (**A**), analysis of P16 expression by Western blot. (**B**), analysis of transcription of *Suv39h2* by quantitative RT-PCR. (**C**), analysis of H3K9 trimethylation (K9me3) within the *p16* promoter by ChIP assays. siR_Ctrl, scramble siRNA control; siR_Suv39h2, siRNA targeted to *Suv39h2*.

Moreover, a rescue assay was carried out to evaluate the role of SUV39H2 in the formation of H3K9me3 within the *p16* promoter induced by CBX7. We found that knockdown of *Suv39h2* expression by siRNA abolished the formation of H3K9me3, and therefore rescued *p16* expression in the tested subclone (CBX7 Clone-2; Figure 7A∼C). Moreover, decrease of H3K9me3 was also observed after knockdown of *G9a* by siRNA, but it could not be detected in the tested cells after treatment with siRNA-Suv39h1, although *p16* expression was increased after these siRNA treatments (Figure S5). These results together indicate that CBX7 may repress *p16* expression by recruiting SUV39H2 to the *p16* 5′UTR to induce the formation of H3K9me3. In addition, in the *p16*-methylated PC3 cells, after stably knockdown of CBX7, *p16* transcription was still not detectable upon further knockdown of *Suv39h2* and/or *Suv39h1* expression by siRNAs. Again, knockdown of *Suv39h1* led to upregulation of *Suv39h2*, whereas knockdown of *Suv39h2* did not lead to upregulation of *Suv39h1* in the PC3 cells (Figure S6).

### Expression alterations of CBX7 and other PRC1 components in human gastric carcinomas

**Figure 8:**
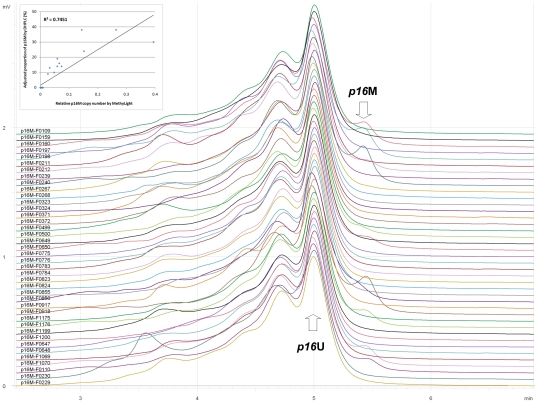
DHPLC chromatogram of methylation status of *p16* CpG island in human primary gastric carcinoma. 20 pairs of gastric carcinomas (with even ID) and their corresponding normal samples (with odd ID) were analyzed by DHPLC. The inserted chart displayed that the result of DHPLC detection correlated with that of quantitative MethyLight analysis well (R^2^ = 0.745; *P* = 0.000).

To validate whether epigenetic silence of *p16* expression correlates negatively with transcription of *Cbx7* and its related components *in vivo*, we first analyzed *p16* methylation status quantitatively by DHPLC and MethyLight in human primary gastric carcinoma samples (GC), the corresponding cutting-edge normal tissues of GC (GCN), and human normal gastric mucosa biopsies from non-cancer patients (*Normal*). Peak for methylated-*p16* (*p16*M) was observed in eleven gastric carcinoma samples: F0110 (14%), F0160 (24%), F0198 (30%), F0212 (10%), F0240 (38%), F0500 (9%), F0650 (13%), F0856 (14%), F0918 (38%), F1070 (16%), and F1176 (19%) (Figure 8). The results were confirmed by MethyLight (Figure 8, inserted chart) and bisulfite clone sequencing (data not shown).

We also analyzed the transcription levels of *p16*, *Cbx7*, and other 6 PcG genes in these samples by quantitative RT-PCR (Table 1). Expression of *p16* in GCs with *p16* methylation was lower than that without *p16* methylation, but not significant (Table 1, *P* = 0.067; the lower ΔCt value, the higher mRNA amount). Transcription of *Cbx7* in GCs was lower than that in GCNs, especially in GCs with *p16* methylation (*P* = 0.009). Transcription of *p16* in GCNs (and GCs) was significantly higher than in *Normal* samples (*P* = 0.003) whereas transcription of *Cbx7* in GCNs (and GCs) was significantly lower than in *Normal* samples (*P* = 0.039), which suggest an inverse relationship between transcription of *p16* and *Cbx7* among gastric samples without *p16* methylation.

**Table 1:** Comparison of transcription levels (ΔCt in qRT-PCR assay, a high ΔCt value means low transcription) of *p16* and seven PcG genes in different kinds of human gastric mucosa samples with and without *p16* methylation.

PcGs	*p16*M a	ΔCt in qRT-PCR (*Mean*±SD)			*P-*value, vs. GCN	
		GC (*n* = 20)^ b^/ c	GCN (*n* = 20)	*Normal* (*n* = 18)	GC d	*Normal*
*p16*	Positive	12.62±2.59^ b^/ e	11.34±1.39 f		0.217	
	Negative	10.42±2.41 c	11.80±2.03	14.63±1.48 g	0.243	**0.003**
	(Total)	11.63±2.41	11.55±2.03		0.919	
*Cbx7*	Positive	8.94±1.08	7.42±1.37		**0.009**	
	Negative	7.77±2.23	6.94±1.42	5.54±1.77	0.364	**0.039**
	(Total)	8.41±1.75	7.2±1.37		**0.019**	
*Ring1*	Positive	6.81±0.82	7.29±1.65		0.359	
	Negative	6.67±1.34	6.57±1.37	6.62±1.38	0.886	0.927
	(Total)	6.74±1.06	6.97±1.53		0.595	
*Bmi1*	Positive	9.21±0.86	9.56±1.29		0.459	
	Negative	8.23±1.66	8.58±0.80	8.46±2.24	0.584	0.764
	(Total)	8.77±1.34	9.12±1.18		0.389	
*Mel18*	Positive	4.81±0.88	5.82±1.35		0.051	
	Negative	4.86±1.10	4.97±1.46	5.80±1.63	0.871	0.198
	(Total)	4.83±0.96	5.44±1.43		0.128	
*Phc2*	Positive	9.21±0.90	9.25±1.33		0.994	
	Negative	8.25±1.77	8.26±1.16	9.33±2.3	0.991	0.213
	(Total)	8.78±1.42	8.8±1.32		0.959	
*Cbx8*	Positive	9.17±0.68	9.38±0.47		0.420	
	Negative	8.50±0.93	9.09±0.72	8.04±1.34	0.149	**0.014**
	(Total)	8.87±0.85	9.25±0.6		0.110	
*Ezh2*	Positive	5.38±1.19	8.90±1.53		**0.000**	
	Negative	6.53±1.73	6.72±1.66	6.61±2.27	0.816	0.885
	(Total)	5.90±1.53	7.92±1.90		**0.001**	

GC: human primary gastric carcinoma sample; GCN: the corresponding cutting- edge tissues of GC; *N*ormal: human normal gastric mucosa biopsies from non-cancer patients;

a methylation of *p16* CpG island (*p16*M) in gastric mucosa tissues from patients with or without cancer;

b GC samples from 11 patients are *p16*M-positive;

c GC samples from 9 patients are *p16*M-negative;

d paired *t*-test;

e *p16*M-positive GCs vs. *p16*M-negative GCs, *P* = 0.067;

f *p16*M was detectable in GCs, but not in the corresponding GCN by DHPLC;

g *p16* mRNA was detectable in 12 of 18 *Normal* gastric biopsies; all of 18 *Normal* samples are *p16*M-negative.

**Figure 9:**
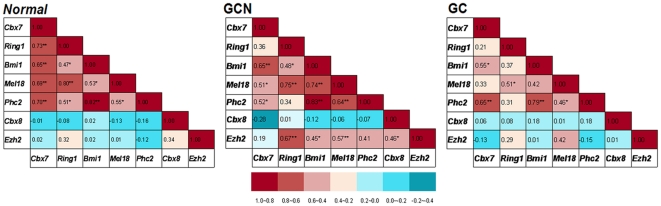
Correlation coefficients between mRNA levels of PcG genes in various kinds of gastric mucosa samples. The relative copy numbers (RCN) was calculated based on the average Ct number of target gene and the *GAPDH* reference [2^-(Cttarget_*gene*-Ct*GAPDH*)^]. The correlation coefficients was calculated based on each gene's RCN for each sample within different groups, including normal gastric mucosa biopsies (*Normal*, *N* = 18), gastric carcinomas (GC, *N* = 20) and their corresponding normal samples (GCN). The significance of correlation coefficients was calculated using the statistic software SPSS16.0 and marked with */** (*P*<0.05/0.01).

Interestingly, in *Normal* tissues from 18 non-cancer patients, we found that transcription of *Cbx7* was positively correlated with those of *Ring1*, *Bmi1*, *Mel18*, and *Phc2*, but not with those of *Cbx8* and *Ezh2* (Figure 9, left chart; *Ps*<0.01). However, in GCN and GC samples from 20 cancer patients, such correlation was progressively disturbed, especially between *Cbx7* and *Ring1/Mel18* (Figure 9, middle and right chart). Moreover, in contrast to *Cbx7* expression, the average expression levels of *Ezh2* in *Normal*, GCs and GCNs were similar in the samples without *p16* methylation, but transcription of *Ezh2* in GCs with *p16* methylation was significantly higher than that in GCNs (*P*<0.000). These results suggest that expression patterns of PcGs, especially CBX7, are changed during development of gastric carcinoma and may affect *p16* expression in the human stomach *in vivo*.

## Discussion

**Figure 10:**
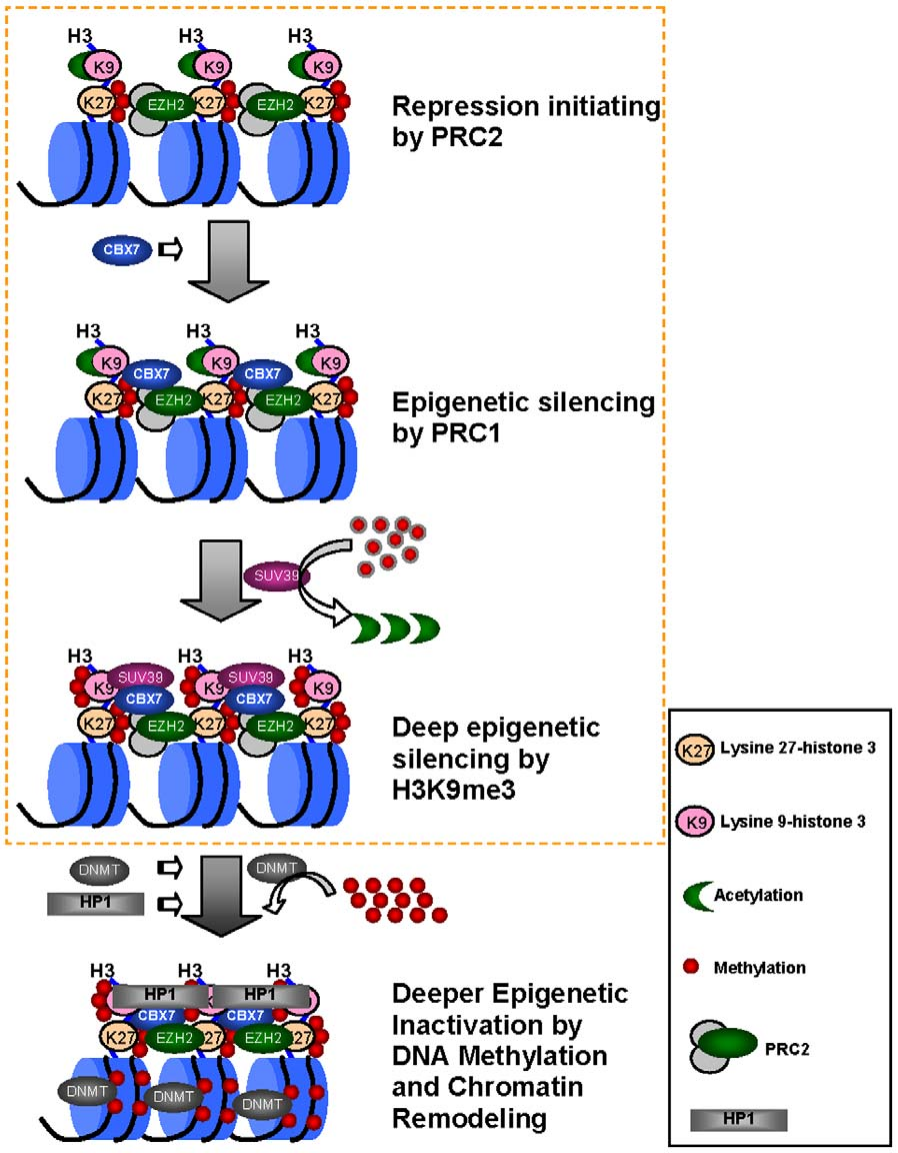
Epigenetic silencing pathway for the *p16-Arf-p15* locus. Gene repression pressure leads to recruitment of EZH2-containing Polycomb repressive complex-2 (PRC2) to the transcription complex, that trimethylates H3K27 and thus initiates the repression. The PRC2-related repression is reversible under condition of transcription pressure. However, in the case of long-term silence, Polycomb repressive complex-1 (PRC1) is recruited to the PRC2-repressed genes and activates H3K9 trimethylation subsequently, which may subsequently induce DNA methylation and chromatin remodeling with assistance of other silencers including DNMTs, HDACs, and heterochromatin binding protein HP1. The content within the yellow dash-square presents the discovery in the present study that connects two epigenetic landmarks the PRC1 binding and H3K9 trimethylation together.

Epigenetic inactivation of *p16* is a well-studied event during carcinogenesis. The detailed mechanism of *p16* inactivation by DNA methylation and histone modification is still unclear. It is well known that H3K9me3 and PRC1 formation in the silenced euchromatic loci, such as the methylated *p16-Arf* locus, are epigenetic inactivation landmarks (Figure 10). Heterochromatin binding protein HP1 can bind with H3K9me3 [13]. Interactions between the PRC1 member CBX7 and H3K9me3 are also observed in *in vitro* peptide pull-down assays [16]. However, it is largely unclear how the locus specific H3K9 trimethylation is initiated during its epigenetic silence processes. In the present study, we first observed, to our knowledge, that the possible H3K9me3 binding protein CBX7 itself could initiate the specific formation of H3K9me3 via HMTase SUV39H2-dependent pathways in the *p16* locus within gastric cancer cell lines.

It is reported that CBX7 promotes proliferation of several human cell lines by repression of *p16* expression [6], [20], [21]. To understand the possible mechanisms through which CBX7 represses *p16* transcription, we established CBX7 overexpression models by transfecting wildtype CBX7 expression vector to MGC803 and BGC823 cells and found both transiently and stably upregulated CBX7 could repress *p16* expression in these gastric cancer cell lines, increase levels of H3K9me3 within the *p16* promoter and RD regions. But an increase of H3K27me3 and H3K9me2 was not detectable within the same regions. CBX7 binding to these regions suggests that CBX7 may repress *p16* expression through initiating or protecting trimethylation of H3K9. Although consistent recruitment of SUV39H2 to the *p16* promoter and 5′UTR regions is not observed in two CBX7 transiently upregulated cell lines, it is found that SUV39H2 is not only upregulated, but also recruited to the *p16* 5′UTR in the CBX7 stably transfected subclones. This indicates that SUV39H2 may be involved in the maintenance of the established H3K9me3 pattern. Like CBX7-GFP, main interaction between CBX7 and SUV39H2 was observed in the Hoechst negative staining area in BiFC assay. Weak interactions of CBX7 with SUV39H2 were also observable in the Hoechst positive staining area. Both are consistent with the distribution of CBX7. These results indicate that CBX7 and SUV39H2 may function together physically within chromatin in the transfected cells. CBX7 could interact with Histone 3.2 or 3.1 directly in BiFC assay [23]. Such interaction between CBX7 and H3.1 in nucleus was also observed in the BiFC assay. Moreover, we found the complex of Myc-CBX7 and YC-H3.1 in Co-IP assay. Unfortunately, we did not find the complex of Myc-CBX7 and YC-SUV39H2 in the Co-IP assay. The lower detection sensitivity of Co-IP compared to that of the BiFC assay and weak interaction between CBX7 and SUV39H2 might account for the result difference. Taken together, it is reasonable to make the assumption that SUV39H2 may responsible for the formation of H3K9me3 at the *p16* promoter. It supports our hypothesis that knockdown of SUV39H2 inhibited the formation of H3K9me3 and rescued p16 expression. Another HMTase G9a, but not SUV39H1, might also contribute to the H3K9 trimethylation. We conclude that CBX7 could initiate or maintain H3K9 trimethylation at the *p16* locus in a SUV39H2/G9a-dependent pattern. Although we did not observe CBX7-induced H3K9me3 formation within the *p15* and *Arf* promoter, we cannot exclude that CBX7 induces trimethylation of other genes in the SUV39H2-dependent way.

CBX7 protein contains a C-terminal Pc-box and a N-terminal chromodomain. The binding of the Pc-box to Ring1 is necessary for the repression of *p16* expression. The chromodomain plays an important role in the binding of CBX7 to H3K9me3 in heterochromatin and silenced euchromatic loci [16], [20], [21]. Mutations in critical residues of chromodomain (F11A and W32A) or deletion of the Pc box (ΔPc) inhibited the ability of CBX7 to extend the life span [20]. In the present study, we found that all of these mutants only partially abrogated the repressive function of CBX7 on *p16* expression, because the residual function of these mutants was still significant. Most importantly, we found that CD mutations or deletion of the Pc-box of CBX7 abolished binding of CBX7 to the tested DNA fragments and greatly disrupted the induction of H3K9me3 within the *p15-Arf-p16* locus in ChIP assays. These results demonstrate that both functional motifs within CBX7 protein contribute to the repression of the *p15-Arf-p16* locus through the H3K9me3 formation.

Mouse CBX7 displays strong affinity for both H3K9me3 and H3K27me3 *in vitro* and is developmentally regulated in its association with chromatin [16], [23]. In the present study, we did not find an increase of H3K27me3 formation within the *p16* promoter. The HMTase EZH2 is responsible for H3K27 trimethylation. Upregulation of *Ezh2* was not observed in the *Cbx7* upregulated cells either. Whether CBX7 binding to H3K27me3 is a prerequisite for recruitment and/or activation of SUV39H2 to the *p16* promoter and subsequent trimethylation of H3K9 is unclear.

Recently, it is reported that enforced upregulation of CBX7 promotes *E-cadherin* expression in 293T cells through its physical interaction with HDAC2 and inhibited its activity within the *E-cadherin* promoter [24]. In contrast, CBX7 could bind to DNMT1 and induce methylation of *E-cadherin* and other tumor suppressor genes, not including *p16*, in the embryonal carcinoma cell line Tera-2 [25]. G9a-DNMT complexes were also detected in colorectal cancer cell line RKO [25]. In the present study, we did not analyze changes of epigenetic modifications within the *E-cadherin* promoter because *E-cadherin* is methylated in both MGC803 and BGC823 cell lines [26]. We analyzed the *p16* promoter and did not find methylation of CpG islands of *p16* in the CBX7 stably transfected cell lines even at passage 80. Instead, H3K9me3 formation was detected in the *p16* 5′ UTR, which may account for the downregulation of *p16* transcription. This difference may be due to the different cell lines used in each study. It also implies that CBX7 have multiple functions and thus regulate gene expression through different mechanisms. Additionally, we also found that stable knockdown of CBX7 by shRNA did not induce de-repression of *p16* expression and demethylation of *p16* CpG island in PC3 cell line either even after *Suv39h2* and/or *Suv39h1* were knockdown by siRNAs. Similar results in RKO cell line were also reported [25]. In consideration of above observation that CBX7 is downregulated in primary gastric carcinomas with *p16* methylation and CBX7 knockdown does not induce demethylation of *p16* in PC3 cells, it is likely that CBX7 upregulation may be sufficient for induction or maintenance of H3K9me3, but not for the establishment of DNA methylation within the *p16* locus. Because of the close relationship between H3K9me3 and initiation of DNA methylation, additional studies will reveal whether CBX7 upregulation is necessary for initiation of DNA methylation of *p16*.

We found that upregulation of CBX7 decreased transcription of *Arf* and *p15*, while downregulation of CBX7 increased transcription of *Arf* and *p15*, which is the same as *p16* gene. Unlike within *p16*, H3K9me3 formation within the *Arf* and *p15* loci was not observed in the tested cancer cell lines after transiently transfection of CBX7. Correlation between *Cbx7* expression and *Arf* methylation/transcription was not observed in the present study either (data not shown). These phenomena imply that CBX7 may favor to repress *p16* more than it does *Arf* and *p15*. It is reported that RD regulates expression of the *p15-Arf-p16* locus simultaneously [5], [22] and we observed H3K9me3 formation at RD region after CBX7 transfection. Thus, formation of H3K9me3 within the RD might account for the downregulation of *p15* and *Arf* transcription induced by CBX7.

Polycomb proteins such as EZH2, BMI1, CBX8, and CBX7 may play important roles in suppressing the expression of the *p16-Arf* locus *in vitro* [6], [7], [12], [27], [28]. However, which kind of PcG protein may contribute to epigenetic silence of this locus *in vivo* is not evaluated yet. Others have reported downregulation of *Cbx7* in human cancer tissues previously [28]–[31]. The relationship between transcription of *Cbx7* and *p16* is not evaluated in gastric tissues. To validate the repression effect of CBX7 on transcription of *p16 in vivo*, the relationships between transcription of *p16*, *Cbx7* and other PRC1 components were analyzed using *Normal* gastric mucosa biopsies from 18 non-cancer patients and paired GCs and GCNs from 20 cancer patients (Table 1). We found that transcription of *Cbx7* was significantly lower in GCs than in GCNs, especially among samples from patients with GCs containing *p16* methylation. Furthermore, we observed that *Cbx7* expression was negatively correlated with *p16* expression: transcription level of *Cbx7* was high in *Normal* tissues with low level of *p16* transcription, but it was low in GCNs and GCs with high level of *p16* transcription. This provides *in vivo* evidence that CBX7 may be involved in the repression of *p16*.

In addition, we also found for the first time that transcription of *Cbx7* was significantly correlated with those of other PRC1 components *Bmi1, Mel18, Ring1*, and *Phc2* in *Normal* gastric tissues, while not with those of *Cbx8* and *Ezh2*. However, such correlations were progressively disturbed in GCNs and GCs.

Downregulation of *Cbx7* in GCs and GCNs may account for disruption of these correlations. It was reported that MEL18 could repress BMI1 in cancer cells [32] and knockdown one of PcGs might release other PcGs from its target gene (such as *p16*) [7]. We proposed that the correlated expression pattern of these PcGs in normal gastric tissues might be necessary for maintenance of their normal functions, and disruption of the pattern might result in abnormal repression of their target genes.

In conclusion, we found that the PRC1 member CBX7 could initiate trimethylation of H3K9 at the *p16-Arf* locus through recruitment and/or activation of the HMTase SUV39H2 to the target locus. This finding links two repressive epigenetic landmarks H3K9me3 formation and PRC1 binding within the silenced domains in euchromatin together, and builds up a full pathway for epigenetic inactivation of *p16* by histone modifications (Figure 10).

## Materials and Methods

### Cell lines and human gastric samples

Human cell lines BGC823, HepG2, MGC803, RKO, and SGC7901 were cultured in RPMI1640 medium with 10% FBS. Human cell line Caski, Colo205, HCT116, HeLa, MHCC97H, Siha, and SW480 were cultured in DMEM medium with 10% FBS. AGS and PC3 were cultured in F12 medium with 10% FBS. All of these cell lines were cultured at 37°C with 5% CO_2_. PC3 cell line was purchased from Cell Line Bank, Chinese Academy of Medical Science. RKO cell line was kindly provided by Dr. Guoren Deng at University of California San Francisco. SW480 and HCT116 cell lines, by Dr. Yuanjia Chen at Peking Union Hospital. HepG2 (from Qingyun Zhang), MGC803, BGC823, SGC7901, HeLa, Caski, Siha, and other cell lines (from Dr. Yang Ke) were obtained from laboratories at Beijing Cancer Hospital/Institute. When the density of cells in the dish was closed to 70%, cells were harvested for extraction of proteins, mRNA, and DNA samples as described below. Proliferation of cells was analyzed with MTT assay.

Twenty paired primary gastric carcinoma (GC) and their corresponding cutting-edge normal tissue (GCN) samples from GC patients (11 males and 9 females, 33–74 years old, the average age 59-y) and 18 gastric normal biopsies (*Normal*) from non-cancer patients (with or without mild/moderate chronic gastritis) were collected and fresh-frozen at −70°C at Beijing Cancer Hospital. All clinical samples and histopathological information for each case were obtained according to approved institutional guidelines.

### Ethics Statement

Informed consent was obtained from all subjects and the institutional review committee approved this study.

### Plasmid construction and transfection

Coding region of CBX7 (GenBank accession no. XM_066324) was cloned into pcDNA3.1(+)/myc-His A vector (Invitrogen) to get the wildtype CBX7 expression vector. To construct mutant vectors, point mutations of caging aromatic residues (F11A and W32A; Figure S2) within the chromodomain of CBX7 and the Pc-box (231–242 amino acids)-deleted CBX7 (ΔPc) were created using Easy Mutagenesis System (FM101; Transgene). These plasmids were transfected to MGC803 and BGC823 cells using Lipofectamine™ 2000 (11668-027; Invitrogen) according to the manufacturer's instruction, respectively.

ShRNA against *Cbx7* was constructed by oligonuleotide targeted to *Cbx7* mRNA (110 nt–128 nt: 5′-caaag tacag cacgt ggga-3′) (accession number XM_066324) in *pGFPU6/Neo* shRNA expression vector including GFP coding sequence (GenePharma Company, Shanghai). The scramble shRNA (5′-gttct ccgaa cgtgt cacgt-3′) was used as negative control. These plasmids were transfected to PC3 cells.

### Generation of the stable cell clones

Wildtype CBX7 expression vector and the control vector were transfected to MGC803 cells. After transfection, cells were cultured in selective medium containing G418 (700 µg/ml). Two subclones (Clone-1 and Clone-2) stably expressing CBX7 and one control cell clone were obtained.

PC3 cells with stable knockdown of *Cbx7* were obtained by sorting twice with FACS as followed. 24 hours after transfection, PC3 cells were cultured in the selective medium containing 300 µg/ml of G418 for 3 weeks. Then, these cells were sorted by FACS. GFP-positive cells were collected and cultured in the selective medium, and sorted again after 3 weeks' culture.

### Protein preparation and Western blot analysis

Total protein was extracted from cultured cells or fresh tissue samples with TRIzol reagent (Invitrogen) according to the manufacturer's instruction. Immunoblotting was performed with the following antibodies: rabbit anti-CBX7 (Ab21873; Abcam), mouse anti-P16 (Ab50282; Abcam), and mouse anti-β-ACTIN (Santa Cruz). Chemiluminescent reagent (Pierce, Thermal) was used to display the protein bands transferred on the PVDF membrane (Millipore).

### RNA extraction, RT-PCR, and quantitative RT-PCR (qRT-PCR) assays

Total RNA was isolated using RNeasy mini kit (Cat. #74104, QIAGEN) according to the manufacture's instruction. Total RNA was reverse transcripted into cDNA using ImProm-II TM Reverse Transcription System (A3800; Promega). Quantitative RT-PCR was performed using power SYBR Green PCR Master Mix (P/N 4367659, Applied Biosystems) on an ABI-7500 Real-Time PCR system (Applied Biosystems). *Cbx7* mRNA level was quantified with TaqMan kit (Roche). The primer sets for the tested genes were listed in Table 2. The relative gene mRNA level was calculated based on the average Ct number of target gene and the *GAPDH* reference [2^-(Cttarget_*gene*-Ct*GAPDH*)^]. SPSS16.0 software was used to analyze the data.

**Table 2:** Primer sequences.

Gene name	Entrez Gene	Assay	Oligo name	Primer Sequence (5′→3′)	PCR Products	Tm (°C)	References
*Cbx7*	23492	RT-PCR	Cbx7-F	aaagtcgagtatctggtgaagtgg	442bp	66	
			Cbx7-R	gctcccgtcgatggctgtgg			
		qRT-PCR	Cbx7-qF	cgtcatggcctacgagga	71bp	54	Pallante et al, 2008
			Cbx7-qR	tgggtttcggacctctctt			
			Cbx7-qProbe	aggaggag			
*Cbx8*	57332	qRT-PCR	Cbx8-qF	ctcgtgaaatggaagggatggt	222bp	53	
			Cbx8-qR	gatgcccctggctgagtcact			
*Bmi-1*	648	qRT-PCR	Bmi1-qF	aattagttccagggcttttcaa	120bp	60	
			Bmi1-qR	cttcatctgcaacctctcctctat			
*Ezh2*	2146	qRT-PCR	Ezh2-qF	cggggatagagaatgtgggttta	173bp	60	
			Ezh2-qR	aggtgggcggctttctttatcatc			
*Mel18*	7703	RT-PCR	Mel18-qF	tggggatggggacaaagagaaaac	200bp	60	
			Mel18-qR	tccgccgccaggggtagat			
*Suv39h1*	6839	qRT-PCR	Suv39h1-qF	ggcaacatctcccactttgt	250bp	56	
			Suv39h1-qR	caatacggacccgcttctta			
*Suv39h2*	79723	qRT-PCR	Suv39h2-qF	cccatctatgaatgcaactcaag	313bp	56	
			Suv39h2-qR	caaaatgagacacattgccgtat			
*G9a*	10919	qRT-PCR	G9a-qF	ctaccgaacagccaagatg	297bp	61	
			G9a-qR	aactgaagaaggcgatgc			
*Phc2*	1912	qRT-PCR	Phc2-qF	aatcctgacgcatgttatcg	275bp	62	
			Phc2-qR	gcttggaacgcttgaacttat			
*Ring1*	6015	qRT-PCR	Ring1-qF	tgggaactgagtctgtatgagc	229bp	56	
			Ring1-qR	tctttcggcaggtaggaca			
*RD*		ChIP-PS1	RD-cF	ccacttatgcagttcctcacc	216bp	56	NT008413.18
			RD-cR	gtcattaaacaggctgaacc			
*p15*	1030	qRT-PCR	p15-qF	agtcaaccgtttcgggaggcg	168bp	62	
			p15-qR	accaccagcgtgtccaggaag			
		ChIP-PS2	p15-cF	ggaacctagatcgccgatgtag	74bp	56	
			p15-cR	tgttttacgcgtggaatgcac			
*p14-Arf*	1029	qRT-PCR	p14-qF	gccaggggcgcccgccgctg	236bp	62	
			p14-qR	ggcccggtgcagcaccacca			
		ChIP-PS3	p14-cF	gtgggtcccagtctgcagtta	61bp	56	
			p14-cR	cctttggcaccagaggtgag			
*p16*	1029	(q)RT-PCR	p16-F	gctgcccaacgcaccgaata	180bp	62	
			p16-R	accaccagcgtgtccaggaa			
		ChIP-PS4	p16-1cF	cggctgggagcagggaggc	155bp	62	Bracken et al, 2007
			p16-1cR	gaatgtggcacccctgaagtcgc			
		ChIP-PS5	p16-2cF	gtccccttgcctggaaagata	154bp	62	Bracken et al, 2007
			p16-2cR	tctccgcagccgccgag			
		MSP-U	p16U-F	ttattagagggtggggtggattgt	234bp	62	Herman et al, 1996
			p16U-R	ccacctaaatcaacctccaacca			
		MSP-M	p16M-F	ttattagagggtggggcggatcgc	234bp	62	Herman et al, 1996
			p16M-R	ccacctaaatcgacctccgaccg			
		DHPLC	p16-uniF	tttttagaggatttgagggatagg	392bp	70→60	Luo et al, 2006
			p16-uniR	ctacctaattccaattcccctacaaacttc			
*Col2A1*			Col2A1-qF	tctaacaattataaactccaaccaccaa	92bp	60	Widschwendter et al, 2004
			Col2A1-qR	gggaagatgggatagaagggaatat			
			Col2A1-qProbe	ccttcattctaacccaatacctatcccacctctaaa			
*GAPDH*	2597	(q)RT-PCR	GAPDH-F	gaaggtgaaggtcggagt	226bp	62	
			GAPDH-R	gaagatggtgatgggatttc			

### DNA preparation, bisulfite treatment, and DNA methylation analysis

Genomic DNA of cell lines and tissue samples was isolated with phenol/chloroform extraction and modified with 5.0 M sodium bisulfite [33]. *p16* methylation was analyzed with MSP, DHPLC, MethyLight, and clone sequencing [34]. Because of PCR bias favoring amplification of the unmethylated templates, the ratio (0.285) of the peak area for the methylated *p16* to that for the unmethylated *p16* in the *p16*-hemimethylated HCT116 cell was used as the constant to adjust the observed ratios for the tissue samples. Primer sets used were listed in Table 2.

### RNA interference assay

The knockdown of *Suv39h2* (sc-106822; Santa Cruz) and *Suv39h1* (sc-38463; Santa Cruz) and *G9a* (sc-43777; Santa Cruz) was accomplished with siRNA duplex transiently. Scramble siRNA (5′-uucuc cgaac guguc acgu-3′) was used as the negative control. Expression level of target gene was analyzed with qRT-PCR and/or Western Blot assays 72 hours after transfection.

### Chromatin Immunoprecipitation Assay (ChIP)

ChIP assays were performed and analyzed essentially as described [35]. The antibodies used were rabbit anti-H3K9me3 (07-625; Upstate), rabbit anti-H3K9me2 (07-441; Upstate), rabbit anit-H3K27me3 (07-449; Upstate), goat anti-SUV39H2 (Ab5264; Abcam), rabbit anti-Myc (Ab9132; Abcam). Rabbit anti-Myc antibody was used to precipitate exogenous CBX7 protein. The enrichment of specific genomic regions was assessed relative to the control IgG (ZB-2301, Zhongshan). Each ChIP experiment was in triplicates and repeated at least three times. Primer sets used were listed in Table 2.

### Confocal Fluorescence Microscopy and BiFC assay

To produce fusion proteins for BiFC analysis, the N-terminal 172 amino acids of Venus (VN) was fused to the N-termini of human CBX7 protein (kindly provided by Claudius Vincenz) [23]. Amino acids 173–238 of YFP (YC, provided by Claudius Vincenz) were fused to the C-termini of SUV39H2. The sequences encoding all fusion proteins were verified and are available upon request. BiFC complexes and co-localization of different PcG proteins in transiently expressing cells were imaged 36 hours after transfection. Cells grown on cover glass were stained with 10 ng/ml Hoechst33342 (Sigma) for 30 min, and fixed in 2% formaldehyde for 10 min at room temperature, washed with phosphate-buffered saline, and mounted for microscopy. The fluorescence images were acquired by using a Leica inverted fluorescence microscope under oil objective. Blank VN- and YC-vector controls were used as negative controls. YC-Histone 3.1 (H3.1, kindly provided by Claudius Vincenz) was used as positive control.

### Co-IP assay

After HEK293 were transfected with the YC-SUV39H2 (or -H3.1) and pcDNA3.1(+)/Myc-His-CBX7 vectors for 48 hours, proteins extracted were immunoprecipitated with mouse anti-Myc antibody (clone 9E10, Clontech) to precipitate exogenous CBX7 protein complexes and separated by SDS-PAGE and immunoblotted with a GFP antibody (ProteinTech Group). The mouse IgG antibody was used as negative control.

## Supporting Information

Figure S1 DHPLC chromatogram of methylation status of *p16* CpG island in human cancer cell lines.
(0.19 MB PDF)

Figure S2 The 3D images of chromodomain of the wildtype CBX7 (PDB id: 2K1B) and its mutant F11A, W32A produced by the DeepView software.
(0.29 MB PDF)

Figure S3 Distribution of CBX7-EGFP and CBX7-SUV39H2 complexes in human gastric carcinoma cell line MGC803.
(0.92 MB PDF)

Figure S4 Images of Co-IP assay for detection of CBX7-Histone 3.1 or CBX7-SUV39H2 complexes.
(0.09 MB PDF)

Figure S5 Effect of siRNA knockdown of HMTases G9a, SUV39H1 and SUV39H2 on formation of H3K9me3 within the *p16* promoter and *p16* expression in the CBX7 stably transfected subclone-2.
(0.20 MB PDF)

Figure S6 Effect of knockdown of *Suv39h2* and *Suv39h1* by siRNA on transcription of *Suv39h2*, *Suv39h1*, and *p16* in PC3 cell line stably transfected with shRNA against *Cbx7* or scramble shRNA control.
(0.46 MB PDF)
