# Supplementary material for: Polycomb CBX7 Directly Controls Trimethylation of Histone H3 at Lysine 9 at the p16 Locus
Source: PLoS One. 2010 Oct 29;5(10):e13732. doi: 10.1371/journal.pone.0013732 (PMC2966406; doi:10.1371/journal.pone.0013732)
Supplement: Figure S1 — DHPLC chromatogram of methylation status of p16 CpG island in human cancer cell lines. (0.19 MB PDF) [file pone.0013732.s001.pdf]

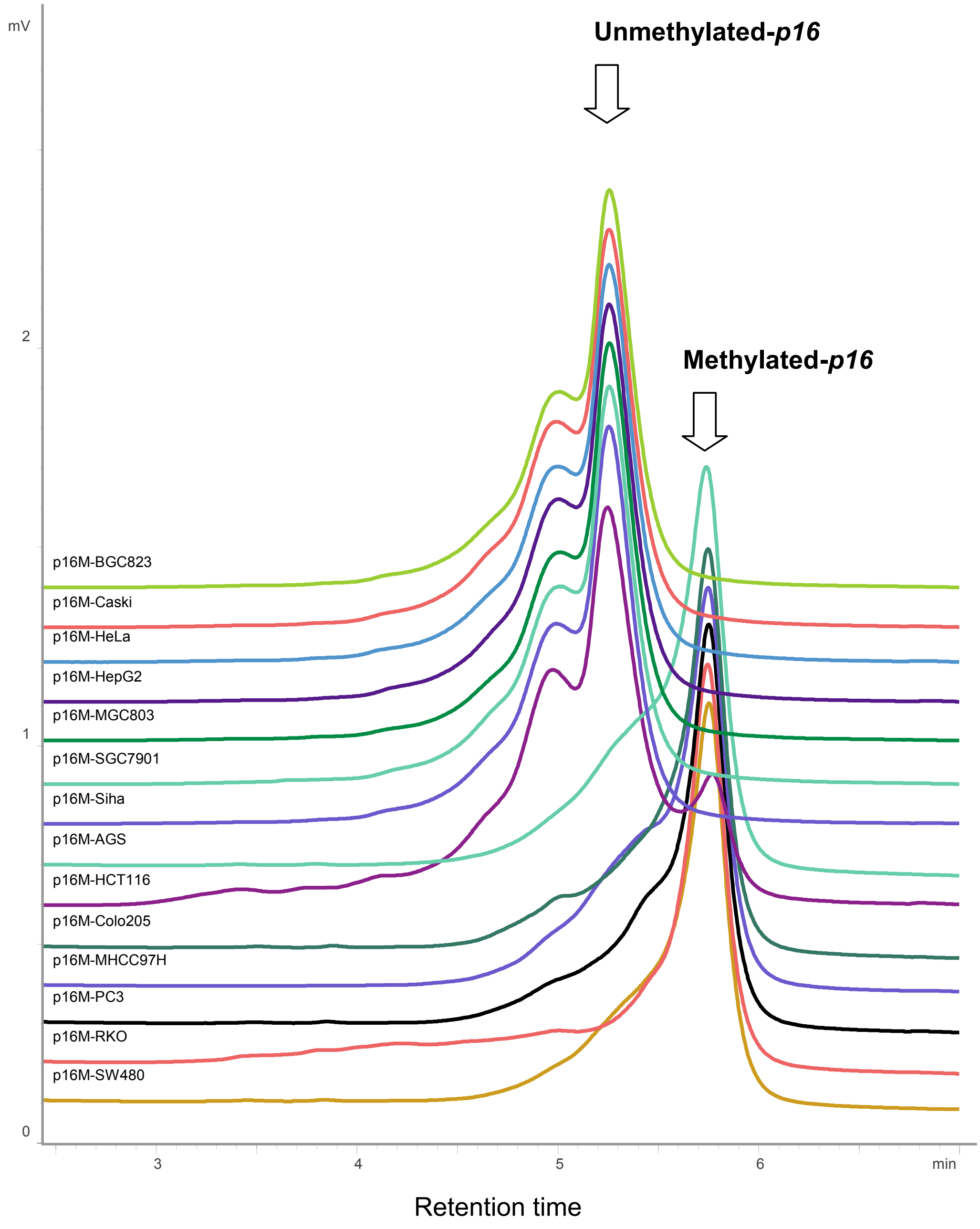

Supplementary Figure S1. DHPLC chromatogram of methylation status of *p16* CpG island in human cancer cell lines. Conditions for bisulfite PCR amplification and DHPLC detection was described previously (Luo et al., 2006). Methylated-*p16* was observed among 6 cell lines: AGS, HCT116, Colo205, MHCC97H, PC3, RKO, and SW480. HCT116 is *p16*-hemimethylated. Ratio of the methylated-*p16* peak area to the unmethylated-*p16* peak area of HCT116 cell was used as the adjusting constant for calculation of proportion of methylated-*p16* alleles in the tested samples showed on Figure 8.
