# Supplementary material for: Polycomb CBX7 Directly Controls Trimethylation of Histone H3 at Lysine 9 at the p16 Locus
Source: PLoS One. 2010 Oct 29;5(10):e13732. doi: 10.1371/journal.pone.0013732 (PMC2966406; doi:10.1371/journal.pone.0013732)
Supplement: Figure S2 — The 3D images of chromodomain of the wildtype CBX7 (PDB id: 2K1B) and its mutant F11A, W32A produced by the DeepView software. (0.29 MB PDF) [file pone.0013732.s002.pdf]

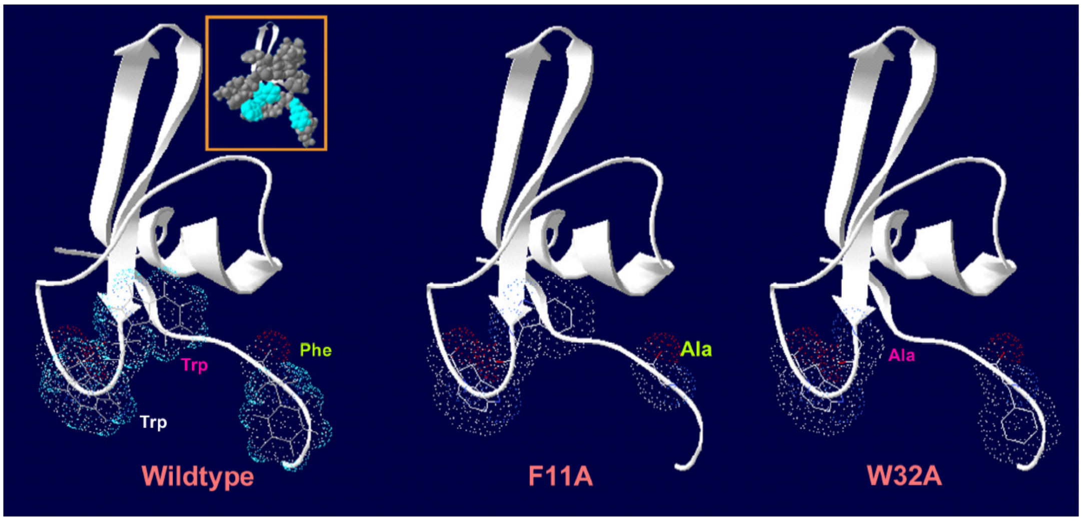

Supplementary Figure S2. The 3D images of chromodomain of the wildtype CBX7 (PDB id: 2K1B) and its mutant F11A, W32A produced by DeepView software. A, amino acid residue alanine (Ala); F, phenylalanine (Phe); W, tryptophan (Trp). Solid 3D view of amino acid residues within the mutation region was also illustrated at the right up corner of wildtype CBX7. The mutation candidates were colored in blue. The 3D structures of these mutants were predicated by the Swissmodel Server Workspace (http://swissmodel.expasy.org/ workspace) using the 2K1BA as the template (Kiefer F, Arnold K, Künzli M, Bordoli L, Schwede T (2009) The SWISS-MODEL Repository and associated resources. Nucl Acids Res 37, D387-D392; Arnold K, Bordoli L, Kopp J, and Schwede T (2006) The SWISS-MODEL Workspace: A web-based environment for protein structure homology modelling. Bioinformatics 22, 195-201; Schwede T, Kopp J, Guex N, and Peitsch MC (2003) SWISS-MODEL: an automated protein homology-modeling server. Nucl Acids Res 31, 3381-3385).
