# Supplementary material for: Polycomb CBX7 Directly Controls Trimethylation of Histone H3 at Lysine 9 at the p16 Locus
Source: PLoS One. 2010 Oct 29;5(10):e13732. doi: 10.1371/journal.pone.0013732 (PMC2966406; doi:10.1371/journal.pone.0013732)
Supplement: Figure S3 — Distribution of CBX7-EGFP and CBX7-SUV39H2 complexes in human gastric carcinoma cell line MGC803. (0.92 MB PDF) [file pone.0013732.s003.pdf]

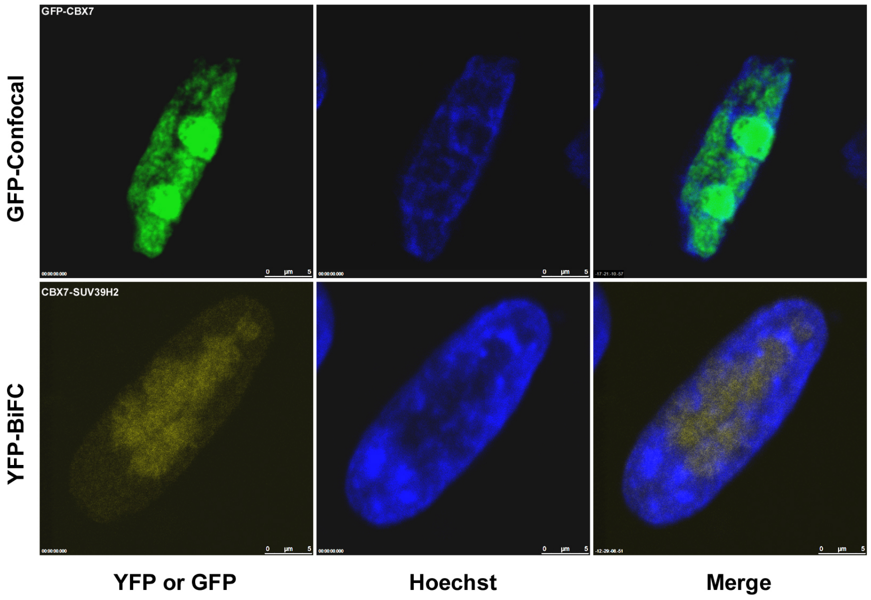

Supplementary Figure S3. Distribution of CBX7-EGFP and CBX7-SUV39H2 complexes in human gastric carcinoma cell line MGC803. At 36 hours after transfection, images of pEFGP-C1-*Cbx7* (green) and Hoechst (blue) fluorescence were obtained with a confocal microscope and merged (top). The confocal images of CBX7-SUV39H2 complexes observed in bimolecular florescence complementation (BiFC) assay (yellow) and Hoechst (blue) fluorescence were obtained and merged (bottom).
