# Supplementary material for: Polycomb CBX7 Directly Controls Trimethylation of Histone H3 at Lysine 9 at the p16 Locus
Source: PLoS One. 2010 Oct 29;5(10):e13732. doi: 10.1371/journal.pone.0013732 (PMC2966406; doi:10.1371/journal.pone.0013732)
Supplement: Figure S4 — Images of Co-IP assay for detection of CBX7-Histone 3.1 or CBX7-SUV39H2 complexes. (0.09 MB PDF) [file pone.0013732.s004.pdf]

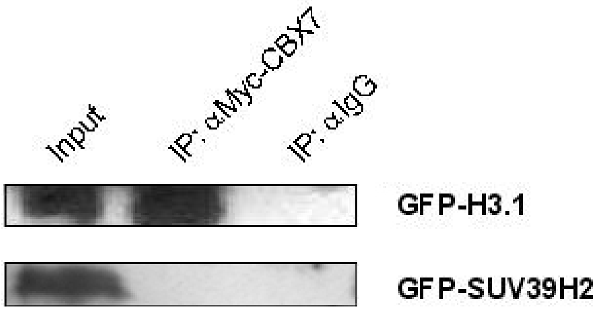


**Supplementary Figure S4. Images of Co-IP assay for detection of CBX7-Histone 3.1 or CBX7-SUV39H2 complexes.** After HEK293 were transfected with the YC-SUV39H2 (or -Histone H3.1) and pcDNA3.1(+)/Myc-His-CBX7 vectors for 48 hours, proteins extracted were immunoprecipitated with mouse anti-Myc antibody to precipitate exogenous CBX7 protein complexes and separated by SDS-PAGE and immunoblotted with a GFP antibody (ProteinTech Group). The mouse IgG antibody was used as negative control.
