# Supplementary material for: Polycomb CBX7 Directly Controls Trimethylation of Histone H3 at Lysine 9 at the p16 Locus
Source: PLoS One. 2010 Oct 29;5(10):e13732. doi: 10.1371/journal.pone.0013732 (PMC2966406; doi:10.1371/journal.pone.0013732)
Supplement: Figure S5 — Effect of siRNA knockdown of HMTases G9a, SUV39H1 and SUV39H2 on formation of H3K9me3 within the p16 promoter and p16 expression in the CBX7 stably transfected subclone-2. (0.20 MB PDF) [file pone.0013732.s005.pdf]

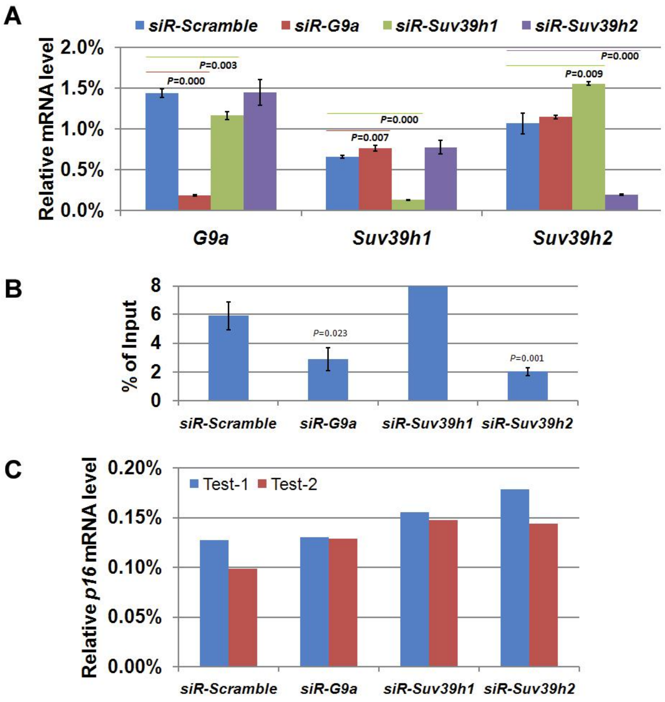

Supplementary Figure S5. Effect of siRNA knockdown of HMTases G9a, SUV39H1 and SUV39H2 on formation of H3K9me3 within the *p16* promoter and *p16* expression in the CBX7 stably transfected subclone-2. (**A**), Transcription of *G9a*, *Suv39h1* and *Suv39h2* was knockdown 72hrs after treatment of *siR-G9a, Suv39h1* and *Suv39h2*, respectively. Transcription of *Suv39h2* was increased by *siR-Suv39h1* treatment significantly. (**B**), comparison of H3K9me3 level within the *p16* promoter by the PS4-ChIP assay 72 hrs after treatment of *siR-G9a, Suv39h1* and *Suv39h2*, respectively. Significant decrease of H3K9 trimethylation was observed compared with the *shR-Scramble* control cells. (**C**), comparison of *p16* mRNA level 72 hrs after treatment of *siR-G9a, Suv39h1* and *Suv39h2*, respectively.
