# Supplementary material for: Polycomb CBX7 Directly Controls Trimethylation of Histone H3 at Lysine 9 at the p16 Locus
Source: PLoS One. 2010 Oct 29;5(10):e13732. doi: 10.1371/journal.pone.0013732 (PMC2966406; doi:10.1371/journal.pone.0013732)
Supplement: Figure S6 — Effect of knockdown of Suv39h2 and Suv39h1 by siRNA on transcription of Suv39h2, Suv39h1, and p16 in PC3 cell line stably transfected with shRNA against Cbx7 or scramble shRNA control. (0.46 MB PDF) [file pone.0013732.s006.pdf]

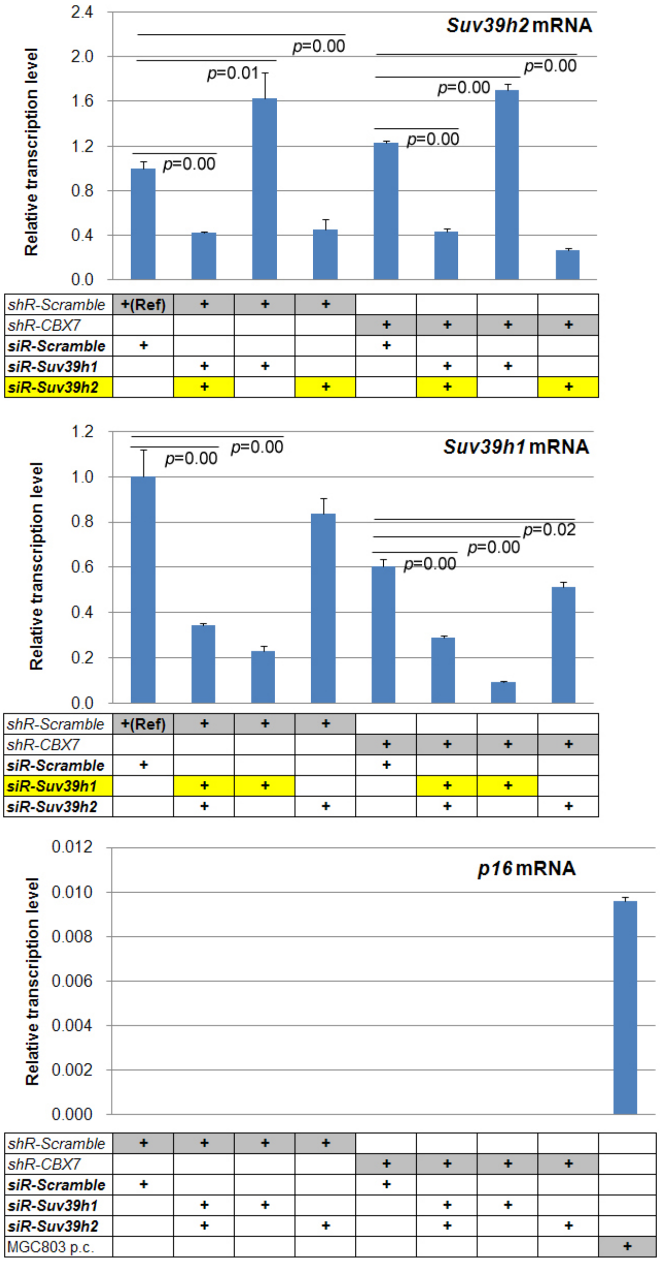

Supplementary Figure S6. Effect of knockdown of *Suv39h2* and *Suv39h1* by siRNA on transcription of *Suv39h2*, *Suv39h1*, and *p16* in PC3 cell line stably transfected with shRNA against *Cbx7* or scramble shRNA control. Transcription of *Suv39h2* and *Suv39h1* was knockdown by *siR-Suv39h2* and *Suv39h1*, respectively. Transcription level of *Suv39h2* was significantly increased by *siR-Suv39h1*, whereas transcription of *Suv39h1* was not increased by *siR-Suv39h2*. Transcription of *p16* was not observed in the PC3 cells, in which *p16* is inactivated by methylation of CpG island, after knockdown of *Suv39h2* and/ or *Suv39h1*. The relative transcription levels of *Suv39h2* and *Suv39h1* were adjusted with their transcription value in the control cells transfected with *shR-Scramble*. MGC803 cells with active *p16* were used as a positive control in the quantitative RT-PCR assay for detection of *p16* mRNA. The adjusting could not carried out for *p16* mRNA because it is not transcribed in the *shR-Scramble* control cells.
